# Editorial: Exploring the health of aquatic organisms through an immune viewpoint

**DOI:** 10.3389/fimmu.2023.1356400

**Published:** 2024-01-09

**Authors:** Antonio Figueras, Lingling Wang, Simon MacKenzie, Jorge Galindo-Villegas

**Affiliations:** ^1^ Institute of Marine Research (IIM), Spanish National Research Council (CSIC), Vigo, Spain; ^2^ Liaoning Key Laboratory of Marine Animal Immunology and Disease Control, Dalian Ocean University, Dailan, China; ^3^ Institute of Aquaculture, Faculty of Natural Science, Stirling, United Kingdom; ^4^ Department of Genomics, Faculty of Biosciences and Aquaculture, Nord University, Bodø, Norway

**Keywords:** AF10 cells, arthropods, CRISPR, crustacea, germ-free, medaka, receptor interacting 9 protein 2, trained immunity

This editorial, emerging from the Frontiers of Immunology Research Topic of the same name, delves into the central theme of immune aspects shaping the health dynamics of aquatic organisms. It synthesizes a series of scholarly investigations within the Research Topic, presenting a comprehensive exploration of immunological nuances in aquatic ecosystems. The curated Research Topic showcases diverse research, from examinations of marine model organisms such as *Exaiptasia pallida* to insights into epigenetic adaptations in invertebrates and mutant fish screening. This scholarly synthesis attests to intellectual diversity and methodological rigor in aquatic immunology, enhancing the comprehension of the intersection between immune responses and the health of aquatic organisms.

Billaud et al. examined the repercussions of climate change on marine ecosystems. Using *Exaiptasia pallida* as a model organism, the authors meticulously scrutinize the immunological responses to heightened temperatures and *Vibrio parahaemolyticus* infections. Observations on morphological changes and heat-shock protein expression underscore the integral role of immune responses in safeguarding the well-being of aquatic organisms. Identifying AF10 cells as potential phagocytes contributes to the nuanced understanding of immune complexities in the aquatic milieu.

Zhao et al. extend the academic narrative by elucidating immune adaptations in arthropods and mollusks. Their exploration of epigenetic modifications facilitating the diversification of immune proteins underscores an evolutionary strategy. Introducing trained immunity puts forward a dynamic dimension where immunological memories endure and transmit across generations. However, the extant limitations in comprehending pathogen-host interactions establish an academic imperative for further elucidation of invertebrate immunity dynamics.

Zhang et al. complement the discourse by examining the Receptor Interacting Protein 2 (RIP2) in the context of viral infections within the orange-spotted grouper. The structural elucidation of EcRIP2 and its purported role in suppressing SGIV replication forms the basis of their analysis. The articulated need for a more comprehensive exploration of downstream signaling pathways brings in a scholarly recognition of the intricate facets governing immune responses in fish viral infections.

Sakaguchi et al. contributed with investigating the symbiotic relationship between host immune systems and intestinal microorganisms in the medaka fish model. The CRISPR-mediated manipulation of interleukin 2 receptor subunit gamma (il2rg) is a focal point, revealing alterations in operational taxonomic units and emphasizing the delicate equilibrium between host immunity and microbial communities. The establishment of germ-free conditions augments the depth of inquiry into the role of microbiota in intestinal health and immune system maturation. Criticisms of methodological limitations acknowledge extant challenges within the academic discourse.

In summary, this Research Topic represents a scholarly endeavor unraveling immune dynamics in aquatic organisms. Each manuscript significantly contributes to our collective understanding, emphasizing the critical importance of adopting an immune perspective. The effectiveness of employing diverse animal models to elucidate specific immune mechanisms governing health, both in vertebrates and invertebrates, is highlighted, aligning with previous suggestions (1). Navigating the intricate landscape of aquatic immunology, these contributions enrich our scientific knowledge and delineate pathways for future research endeavors. They underscore the imperative of fortifying the health and resilience of aquatic ecosystems through a comprehensive understanding and appreciation of the nuanced complexities inherent in immune responses.

## Author contributions

AF: Conceptualization, Writing – original draft. LW: Conceptualization, Writing – original draft. SM: Conceptualization, Writing – original draft. JG-V: Conceptualization, Visualization, Writing – review & editing, Supervision.
